# Three-Dimensional Navigated Transsacral Screw Fixation After Failed Conservative Treatment in Patients with Sacral Insufficiency Fractures: A Retrospective Observational Study with a Three-Month Follow-Up

**DOI:** 10.3390/jcm14113749

**Published:** 2025-05-27

**Authors:** Philipp Mantilla-Mayans, Diego A. Sandoval-Lopez, Juan M. Lopez-Navarro, Karen Velazquez, Marcos A. Suarez-Gutierrez, Arnulfo Garza-Silva, Saeed Yassin, Vasilis Karantzoulis, Karla Silva-Hernández, Edgar Santos, Farzam Vazifehdan

**Affiliations:** 1 Spine Center Stuttgart, Paulinenhilfe, Diakonie-Klinikum Stuttgart, Rosenbergstrasse 38, 70176 Stuttgart, Germany; philipp.mantilla-mayans@diak-stuttgart.de (P.M.-M.); diego.alberto.sandoval.lopez@uni-oldenburg.de (D.A.S.-L.); yassinsaeed86@gmail.com (S.Y.); edgar.santos@diak-stuttgart.de (E.S.); 2 Department of Neurosurgery, Carl von Ossietzky University of Oldenburg, 26129 Oldenburg, Germany

**Keywords:** sacrum, pelvic fractures, osteoporotic fractures, aged, image-guided surgery, internal fracture fixation, postoperative pain, length of stay

## Abstract

**Objectives**: This study evaluated the effectiveness of three-dimensional (3D) navigated transsacral screw fixation in patients with sacral insufficiency fractures (SIF) who experienced inadequate mobilization after conservative management. **Methods**: We conducted a retrospective analysis of 53 patients (mean [±standard deviation] age 78.7 [±10.8] years; range 43.7–92.4; 81.1% female) with sacral insufficiency fractures confirmed by computed tomography or magnetic resonance imaging. Documented osteoporosis was present in 28 (52.8%) of these patients. All had failed conservative management due to persistent sacral pain or inability to mobilize. Therefore, they underwent 3D-navigated transsacral screw fixation. We collected data on demographics, fracture classification (FFP system), pain levels (Visual Analog Scale [VAS]), opioid consumption, time to mobilization, and length of hospital stay. Clinical outcomes were recorded at discharge, 1 month, and 3 months post-op; telephone interviews were conducted between 1 January and 28 February 2024 to assess longer-term pain relief and functional status. **Results**: Using the fragility fractures of the pelvis (FFP) classification, 60.8% of patients had FFP IIa fractures, 11.8% had FFP IIc, and 21.6% had FFP IVb. Two transsacral screws were placed in 34.0% of cases and three in 38.0%. We observed significant postoperative pain reduction—median (interquartile range [IQR]) VAS-at-rest decreased from 5 (4) preoperatively to 2 (2) at discharge and 0 (1) at 3 months (*p* < 0.001)—along with reduced opioid use (from 92.5% of patients on the immediate postoperative day to 45.0% at 3 months, *p* = 0.003). Early mobilization was achieved in 96.2% of patients. The mean hospital stay was 11.7 ± 5.1 days (95% confidence interval [CI]: 10.3–13.2; range 3–25 days), with the few outlier cases (>21 days) attributable to medical complications or delayed rehabilitation placement. The overall complication rate was low (7.5%, predominantly minor wound issues), and the 3-month reoperation rate was 7.7%. **Conclusions**: The 3D-navigated transsacral screw fixation is a minimally invasive and effective approach for managing sacral insufficiency fractures. It provides substantial pain relief, enables early mobilization, and demonstrates a low complication rate at 3 months. This technique shows promise in improving short-term outcomes for patients who do not respond to conservative care, regardless of osteoporosis status, although further research is needed to evaluate long-term fracture healing and functional recovery.

## 1. Introduction

Sacral insufficiency fractures (SIF) are stress fractures that occur when normal physiological loads exceed the strength of structurally compromised sacral bone. Although first recognized by Lourie et al. in 1982 [1], the diagnosis of SIF has become more frequent with the widespread adoption of magnetic resonance imaging and CT. Recent estimates place the incidence of SIF at approximately 1–1.8% among at-risk populations, particularly older adults with low bone density undergoing pelvic imaging [2,3]. This foundational work laid the groundwork for subsequent research and understanding of SIF. Already weakened bones are particularly vulnerable to insufficiency fractures, and one main factor appears to be mechanical overstressing [4]. Patients typically present with persistent and immobilizing pain in the lower back and dorsal pelvic ring [2]. Magnetic resonance imaging (MRI) is widely regarded as the most sensitive modality for detecting sacral insufficiency fractures, since fluid-sensitive sequences reveal reactive bone marrow edema and subtle fracture lines that often remain occult on plain films and even on CT [5,6,7]. In contrast, computed tomography (CT), though less sensitive for early marrow changes (60–75% sensitivity), provides high-resolution cortical detail and is invaluable for confirming fracture presence and delineating its exact configuration once a lesion is suspected [8].

Treatment options for SIF fall into three broad categories: conservative management, percutaneous cement augmentation (sacroplasty), and surgical fixation. Conservative treatment (bed rest, analgesics, and osteoporotic medications) can sometimes yield fracture healing, but prolonged immobilization often leads to complications and persistent pain. Sacroplasty, the percutaneous injection of polymethylmethacrylate (PMMA) bone cement into the fracture zone, has emerged as a minimally invasive intervention that provides rapid pain relief [9]. A recent systematic review by Briggs et al. showed sacroplasty achieves superior pain reduction compared to conservative management and even to traditional screw fixation [10]. However, PMMA augmentation is not without risk. Cement leakage is relatively common (reported in up to ~27% of cases) and can injure adjacent nerves; for example, leakage into the sacral foramina has caused L5 nerve root radiculopathy [11]. These concerns are echoed in the study by Schwetje et al., which discusses the risks and benefits of balloon-assisted sacroplasty [4]. The exothermic reaction of curing cement can also result in thermal necrosis of surrounding bone or neural elements [12]. Although major complications of sacroplasty are rare (approximately 0.3% requiring surgical intervention in a large meta-analysis), these potential issues warrant caution [13]. Indeed, catastrophic systemic effects like bone cement implantation syndrome have been reported in arthroplasty patients [11], underscoring the need for careful patient selection and technique during cement injections.

Recently, the surgical techniques have shifted to a more minimally invasive variant. Fuchs described a navigated technique for transsacral screw fixation [14], and Balling makes a didactic description of this technique using modern navigation technology [2]. However, there are concerns about bone quality and screw hold in osteoporotic bone [4,15]. Three-dimensional navigated screw fixation offers precise instrument placement and multi-cortical purchase even in osteoporotic bone. This addresses concerns about poor screw hold with standard methods in fragility fractures. Importantly, navigated fixation provides internal stabilization of the pelvis without the use of cement, avoiding the aforementioned risks of sacroplasty. Early reports suggest that navigated transiliac–transsacral screws can achieve high accuracy and safety [16,17,18]. However, data on clinical outcomes (pain relief, mobility improvement, etc.) for navigated screw fixation in SIF remain limited, and comparisons between surgical fixation and sacroplasty are still being explored.

This study aims to evaluate the 3-month outcomes of patients with unilateral or bilateral sacral insufficiency fractures (SIF) undergoing 3D-navigated transsacral screw fixation after failed conservative management. Specifically, we evaluated whether 3D-navigated transsacral screw fixation could effectively reduce pain and shorten hospital stay, thereby improving early mobilization. We also sought to document complications and any need for reintervention within the short term. By examining a consecutive series of such patients, this study provides insight into the feasibility and benefits of navigation-assisted fixation as an alternative to cement augmentation or prolonged nonoperative care.

## 2. Methods

**Figure 1:**
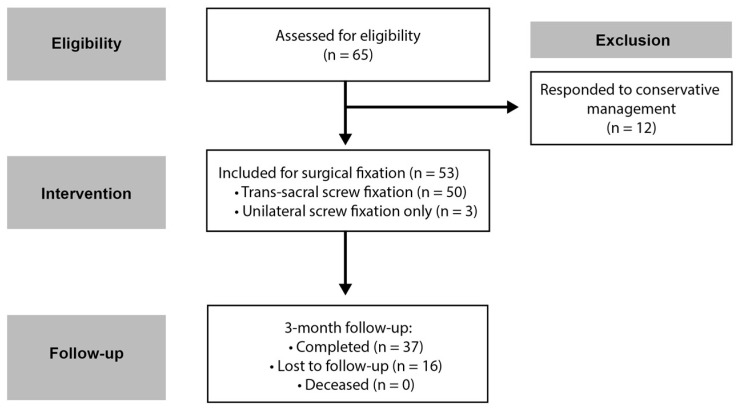
STROBE-compliant flow diagram outlining patient selection, exclusion, intervention and follow-up. Sixty-five patients with sacral insufficiency fractures (SIF) were assessed for eligibility; twelve patients responded to conservative management and were therefore excluded. The remaining fifty-three patients failed conservative treatment and underwent 3D-navigated sacral screw fixation (study cohort)—of these, fifty received transsacral screws crossing both sacral alae, and three had unilateral fixation only due to anatomical constraints. At the 3-month follow-up, thirty-seven patients had complete data, sixteen were lost to follow-up, and none were deceased.

The following is an observational retrospective study conducted on 53 patients with sacral insufficiency fractures treated with 3D-navigated transsacral screw fixation between 1 January 2020 and 31 December 2023 at our institution. This study was conducted in accordance with the Declaration of Helsinki and was approved by the Ethics Committee of the Landesärztekammer Baden-Württemberg (approval No. F-2015-085, approved on 16 July 2020); informed consent was obtained from all participating patients. This study adhered to the Strengthening The Reporting Of Observational Studies (STROBE) reporting guidelines [19]. A STROBE-compliant flow diagram of patient inclusion and exclusion is provided (Figure 1).

### 2.1. Inclusion and Exclusion Criteria

Inclusion criteria were patients diagnosed with sacral insufficiency fractures, typically affecting patients over 65 years old (though no strict age threshold was applied in this study), confirmed by computed tomography (CT) or magnetic resonance imaging (MRI) who had failed initial conservative management due to inadequate mobilization or persistent pain and were treated with the 3D image-guided transsacral screw fixation method. Inadequate mobilization was defined as an inability to ambulate due to persistent sacral pain despite analgesic therapy. Exclusion criteria were grossly unstable pelvic ring injuries requiring spinopelvic fixation and those exhibiting significant neurological deficits necessitating decompression rather than stabilization. Data were collected from digital medical records, and follow-up evaluations were planned at one and three months. Figure 1 shows a diagram outlining patient selection, treatment allocation, and follow-up.

### 2.2. Variables

Variables regarding medical history included sex, age (years), body mass index (BMI), presence and number of comorbidities, presence of minor trauma (defined as ground-level fall, repetitive stress or microtrauma, sudden twisting or violent movements, minor trauma in vehicular accidents, or lifting heavy objects), duration of symptoms (days), and type of Fragility Fracture of the Pelvis (FFP) according to the Rommens classification as assessed per MRI or CT imaging [20]. FFP type I involves an isolated anterior pelvic-ring fracture; FFP II includes nondisplaced sacral (posterior) fractures (with or without anterior involvement); FFP III denotes a unilateral displaced posterior fracture; and FFP IV indicates bilateral posterior fractures with complete pelvic instability. Surgical variables included the number of spinal levels operated on, the total number of transsacral screws, the total number of unilateral screws, the need for screw correction after surgery, the duration of surgery (minutes), surgery complications, the start of mobilization after surgery (day), hospital stay (number of days), the presence of prolonged hospitalization (in accordance with prior research [21], prolonged hospitalization was defined as a stay exceeding 21 days), screw alterations at 3 months post-op (absent, presence of loosening or backed out), and the need for reintervention at 3 months. Assessment of pain through the Visual Analog Scale (VAS) was performed pre- and postoperatively, at the first month after surgery and after the third month. Moreover, the requirement of opioids, presence of paresis the post-surgery period, and Cumulated Ambulation Score are also taken into consideration [22].

### 2.3. Treatment Protocol

All patients initially received conservative treatment, including physical therapy with permission for full weight-bearing and analgesics as needed. Surgery was offered to those in whom mobilization was inadequate or who failed conservative treatment, as defined above. The surgical approach involved 3D-navigated transsacral screw fixation, irrespective of anterior fracture fixation, and without additional posterior fixation (e.g., spinopelvic fixation). All procedures were performed by two senior orthopedic spine surgeons with over 10 years of experience in lumbopelvic fixation, each well-versed in navigated sacropelvic techniques. This ensured consistency in the surgical technique across all cases.

### 2.4. Surgical Procedure

Under general anesthesia, two small incisions were made over the superior posterior iliac spine to allow installation of the navigation reference frame on the posterior iliac crest using Schanz screws. A 3D fluoroscopy scan (Cios Alpha, Siemens Healthineers, Erlangen, Germany) was performed, and the data were transferred to the navigation unit (Curve Navigation System; Brainlab AG, Munich, Germany) for real-time 3D navigation. Suitable transsacral trajectories were identified, and approximately 3 cm skin incisions and blunt soft tissue dissections were carried out. Screw lengths were determined using BrainLab’s virtual measuring tool and verified with preoperative CT scans.

**Figure 2:**
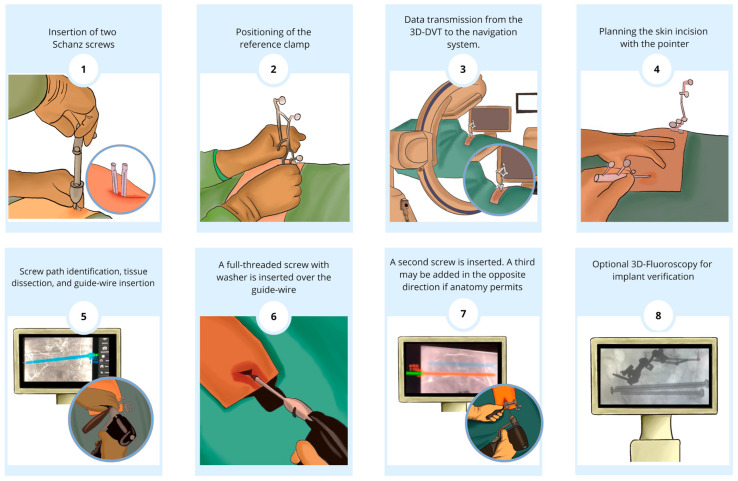
Summarized Procedural Steps (after preoperative diagnosis and classification). (**1**,**2**) Insertion of two Schanz screws and positioning of the reference clamp; (**3**) intraoperative 3D-DVT with C-arm (Siemens Cios) and data transmission to the navigation system (BrainLab); (**4**,**5**) planning of the skin incision with the pointer, identification of the screw trajectory, blunt soft tissue dissection (~3 cm incisions), and insertion of the guide-wire (ensuring it was 2–3 cm shorter than the screw to prevent forward movement); (**6**,**7**) sequential S1 to S2 transsacral screw insertion (first screw: fully threaded screw with washer inserted along the guide-wire, usually without overdrilling; second screw: inserted subsequently, with an optional third screw placed in the opposite direction to prevent collision between screw heads if the anatomy allows); and (**8**) optional postoperative 3D fluoroscopy for implant verification.

Fracture fixation was tailored to the injury pattern in the following ways: Bilateral unstable fractures (e.g., FFP type IVb) were treated with two transsacral screws at S1 (one from each side) plus an additional screw at S2 when needed (for a total of three screws). Unilateral fractures (e.g., FFP IIa) were addressed with a single-sided approach, using one or two screws across the fracture depending on stability requirements. Using a navigated drill sleeve, guide wires were inserted through both sacro-iliac joints and the sacrum at the S1 level. X-ray imaging with 2D fluoroscopy confirmed proper guide wire placement, after which transsacral screws with washers were inserted. This process was repeated for a second screw at the S2 level, and in most cases, a third screw at S1. The third screw was placed contralaterally in cases of bilateral sacral massa lateralis fractures or ipsilaterally when necessary. Guide wires were removed, the wounds were irrigated, and the incisions were closed. Navigation tools were extracted, and the implant position was confirmed intraoperatively via navigation and optional 3D-fluoroscopic scan and again on postoperative pelvic radiographs (AP, inlet, and outlet views) prior to discharge. In addition to transsacral screw fixation, supplemental unilateral sacro-iliac screws were used when needed to optimize construct stability or when patient anatomy precluded safe transsacral trajectories. In three patients, dysmorphic sacral corridors did not allow passage of a transsacral screw without undue risk to neurovascular structures; these were therefore stabilized exclusively with one to three unilateral sacro-iliac screws. The procedural steps are visually summarized in Figure 2 and shown in the Supplementary Video S1.

### 2.5. Postoperative Care and Follow-Up

**Figure 3:**
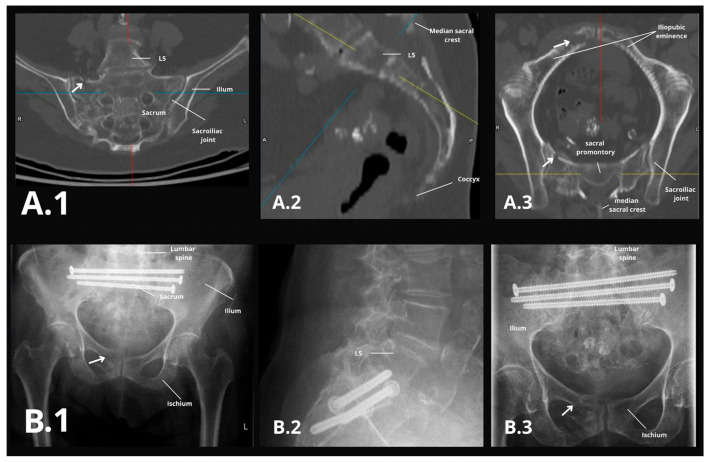
Preoperative and postoperative imaging. (**A**) The firs row shows preoperative axial pelvic CT scan demonstrating a type IIIc sacral insufficiency fracture (Fragility Fractures of the Pelvis [FFP] classification). (**A.1**) Coronal CT image showing a sacral fracture. The white arrow indicates the fracture line through the sacrum. (**A.2**) Sagittal CT reconstruction and (**A.3**) axial CT image of the pelvis showing the extent of the fracture. (**B**) The second row shows postoperative radiographies showing transsacral screw fixation with two screws at S1 and one screw at S2 levels. (**B.1**) Anteroposterior (AP) pelvic X-ray, (**B.2**) lateral lumbar spine X-ray, and (**B.3**) AP pelvic X-ray showing fixation of the sacrum after 1 month of follow-up. Blue, red and yellow lines have no semantic significance.

Postoperatively, patients were allowed full weight-bearing under physiotherapist supervision beginning on postoperative day one, typically using a walker or crutches for the initial weeks. Follow-up evaluations at 4 weeks and 3 months postoperatively included clinical assessment and anteroposterior and lateral X-rays to verify screw position and detect any screw loosening (e.g., implant back-out or radiolucent halos around screws). Any evidence of loosening or migration was recorded, and revision was considered if indicated, as shown in Figure 3B. CT scans were reserved for cases with residual or new pain (Figure 3A). Antiosteoporotic therapy was initiated or optimized for all patients according to DVO guidelines (Dachverbandes Osteologie e.V.). Pain levels, adverse events, and opioid usage were recorded during routine clinical rounds and documented digitally. Patients whose 3-month visit occurred after 31 December 2023 were contacted by telephone between 1 January and 28 February 2024 to complete their 3-month outcome assessment.

### 2.6. Data Analysis

Continuous variables are presented as mean and standard deviation (SD) for normally distributed data or median with interquartile range (IQR) for non-normally distributed data, assessed via the Shapiro–Wilk test. Categorical data were presented as frequencies and percentages. Paired comparisons (pre-/postoperative outcomes) employed Student’s *t*-test (normal data) or Wilcoxon signed-rank test (non-normal). Longitudinal pain scores (VASs) were analyzed with the Friedman test, post hoc multiple comparisons were made with Wilcoxon tests and corrected with Bonferroni, and opioid use with the Cochran’s Q test. Predictors of a prolonged hospital stay (defined as >21 days, roughly the 90th percentile of our cohort’s hospital length of stay) were assessed via logistic regression. Only complete cases were included in the inferential analyses. A *p* < 0.05 defined statistical significance. Analyses used SPSS 20.0 (IBM Corp., Armonk, NY, USA) and R version 4.4.1 (R Foundation for Statistical Computing, Vienna, Austria) for the development of graphs.

## 3. Results

A total of 53 patients were included, with a mean age of 78.7 ± 10.8 years (range 43.7–92.4). The majority (81.1%) were female. By using the Fragility Fractures of the Pelvis (FFP) classification [20], it was determined that the most common type of fracture was FFP IIa at 60.8% (n = 31) and 21.6% (n = 11). No cases of FFP IIb, IIIa, IVa, or IVc were observed. Nearly half of the patients (46%, 23 of 50 documented cases) reported a history of minor trauma (low-energy falls or strain) precipitating the fracture. Comorbid conditions were common: 52.8% of patients had a known diagnosis of osteoporosis, reflecting their high fracture risk. Besides osteoporosis, the most common comorbidity was hypertension, with an equal proportion of 52.8%. On average, patients had multiple co-existing illnesses—the median number of comorbidities was 5 (IQR 5, range 1–16), underlining the frailty of this population. The median duration of symptoms (from onset of pain to surgery) was 16 days (IQR 34, range 1–274), although two patients’ symptom durations were unknown. Baseline demographic and clinical characteristics are summarized in Table 1.

### 3.1. Surgical Characteristics

Surgical fixation targeted the S1 + S2 levels in 98.1% (n = 52) of cases, with one patient receiving an isolated S1 screw. All 53 patients underwent navigated percutaneous screw fixation. In 50 patients (94.3%), at least one transsacral screw was successfully placed across the sacrum from one ilium to the other. Three patients (5.7%) did not receive a transsacral screw due to unfavorable sacral anatomy (as determined intraoperatively by navigation); they were instead treated with unilateral sacro-iliac screws for stabilization. Among the 50 cases with transsacral fixation, 18 patients (34.0% of the cohort) received two transsacral screws, and 20 patients (37.7%) received three screws. The remaining 12 patients (22.6%) had a single transsacral screw placed (typically for a unilateral fracture pattern). No surgeries were terminated prematurely due to complications such as vascular injury or excessive intraoperative blood loss. Procedural details, including screw configurations, are presented in Table 2. In two cases, intraoperative 3D scans revealed suboptimal initial screw trajectories, and these screws were repositioned during the same surgery; no patient required a return to the operating room for screw malposition. All patients had their screw positions documented by the intraoperative 3D scan, and proper placement was confirmed in every case.

### 3.2. Pain and Functional Outcomes

**Figure 4:**
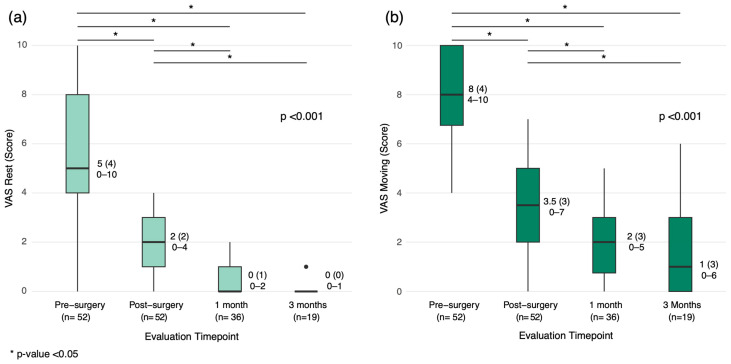
(**a**) Visual Analog Scale (VAS) scores of rest at different evaluation timepoints. (**b**) VAS scores during mobilization at different evaluation timepoints. Data are presented as median (interquartile range) and range (min–max); *p*-value was determined in complete cases (n = 17) using the Friedman test for related samples in a two-factor analysis of variance by ranks. Multiple comparisons were performed post hoc using the Wilcoxon signed-rank test with Bonferroni correction.

Of the 53 patients, 37 had pain scores available at the 3-month follow-up; however, only 17 patients (32%) completed all scheduled VAS assessments at every timepoint from preoperative to 3 months. Thus, the longitudinal pain analysis was conducted on the complete cases. Preoperative median (IQR, range) Visual Analog Scale (VAS) scores were 5 (4, 0–10) at rest and 8 (4, 4–10) during mobilization, indicating significant immobilizing pain in most patients. Median (IQR, range) VAS scores at rest dropped to 2 (2, 0–4) and during mobilization to 3.5 (3, 0–7). At one month, VAS scores further decreased to 0 (1, 0–2) at rest and 2 (3, 0–5) during mobilization. By three months, scores were 0 (0, 0–1) at rest and 1 (3, 0–6) during mobilization, reflecting a 75% overall reduction in pain levels (*p* < 0.001; Figure 4).

Opioid use increased slightly on postoperative day one (92.5%) but declined significantly thereafter, with 51.4% (n = 19) using opioids at one month and 45.0% (n = 9) at three months (*p* = 0.003). Early mobilization was achieved in 96.2% (n = 51) of patients on postoperative day one, with two patients requiring delayed mobilization (≥day 2). Mobility levels, as assessed by the Cumulated Ambulation Score, remained stable at a median (IQR, range) of 2 (1, 0–3) preoperatively and postoperatively, with no significant changes over three months. Detailed functional recovery metrics are provided in Table 3.

### 3.3. Hospital Stay and Complications

The mean (SD) hospital stay was 11.7 (5.1) days (95% CI: 10.3–13.2, range: 3–25 days). A simple logistic regression identified osteoporosis (OR: 2.9, 95% CI: 1.2–7.1, *p* = 0.02) and longer symptom duration before surgery (OR: 1.7, 95% CI: 1.0–2.9, *p* = 0.04) as independent predictors of prolonged hospitalization.

The overall complication rate was 7.6% (n = 4), with the main complication being minor wound infections (5.7%). Screw alterations occurred in 13.5% of cases (n = 7), where screw back-out was the main alteration with 9.4% (n = 5) of patients. At three months, the reoperation rate was 7.7% (n = 4). No cases of cerebrospinal fluid leaks or new neurological deficits were observed. Table 4 shows the hospital stay days and complication characteristics.

## 4. Discussion

### 4.1. Overview of Study and Clinical Implications

This study demonstrates that 3D-navigated transsacral screw fixation is a safe, effective, and minimally invasive surgical option for patients with sacral insufficiency fractures. The findings reveal significant improvements in pain management, early mobilization, and a low complication rate, reinforcing the technique’s pivotal role in modern fracture management.

We observed a striking 75% reduction in median pain scores at three months (*p* < 0.001), with Visual Analog Scale (VAS) ratings falling from 5 at rest and 8 during mobilization preoperatively to 0 and 1, respectively. Opioid use likewise dropped from 90.4% preoperatively to 45% at three months (*p* = 0.003), highlighting the role of surgical stabilization in reducing long-term analgesic requirements. While sacroplasty has been shown to deliver rapid pain relief in SIF patients, our 3D-navigated screw fixation achieved comparable early pain control and conferred immediate mechanical stability. Notably, median VAS at one month was 0, similar to published sacroplasty outcomes, and 96% of patients were ambulating by postoperative day 1, an early mobilization benefit directly attributable to the robust fixation provided by the screws.

Functional recovery metrics were equally notable, with 96.2% of patients mobilizing on the first postoperative day and 92% achieving functional independence within one month, which increased to 96% by three months. The mean (SD) hospital stay was 11.7 (5.1) days, with osteoporosis and prolonged symptom duration identified as predictors of extended hospitalization.

Surgical outcomes highlighted the feasibility of achieving robust multi-point cortical fixation even in osteoporotic bone. S1 + S2 fixation was performed in 98.1% of cases, with three screws placed in 37.7% of patients and two screws in 32.1%. The median (IQR) surgical duration of 55 (24) minutes compared favorably with traditional open procedures, underscoring the efficiency of the 3D-navigated approach.

### 4.2. Advantages of 3D-Navigated Fixation

This study underscores several critical advantages of 3D-navigated transsacral screw fixation over conventional fluoroscopy-guided methods. Enhanced precision, achieved through real-time navigation, significantly reduces the risk of screw malpositioning and ensures secure cortical engagement, even in osteoporotic bone. This precision directly translates to superior clinical outcomes, as evidenced by the reduction in pain and early functional recovery observed in this cohort.

The minimally invasive nature of the approach, characterized by smaller incisions, was associated with reduced soft tissue trauma, minimal intraoperative blood loss, and shorter recovery times. Furthermore, patients demonstrated early rehabilitation potential, with 96.2% mobilizing on the first postoperative day. This rapid return to mobility underscores the structural stability afforded by the fixation technique and its role in promoting accelerated functional recovery. This is supported by Regenbogen et al., who reported excellent short-term outcomes and low complication rates using a 3D-navigated transsacral bar in patients over 65 years old [23].

An additional, often overlooked benefit of 3D navigation lies in its capacity to substantially lower radiation exposure for both patients and surgical teams. By reducing reliance on fluoroscopy, 3D navigation mitigates the risks associated with cumulative radiation exposure, as previously corroborated by Takao et al. and Vaishnav et al. [24,25]. This reduction enhances the safety profile of the surgical environment and aligns with broader efforts to minimize iatrogenic risks.

It should be noted that there is a learning curve for adopting 3D-navigated screw fixation. Surgical teams already proficient in the technique, like ours, may achieve low complication rates and efficient workflow. In contrast, centers new to navigation may initially encounter longer operative times or higher technical error rates. As experience grows, the precision and benefits of navigation become more consistent, as reported in multi-center studies [17].

### 4.3. Contextualization with Existing Literature

The findings of this study align with and extend the growing body of evidence supporting minimally invasive techniques for SIF. While sacroplasty has been shown to provide substantial pain relief, as reported by previous studies [15,26], the current study demonstrates that 3D-navigated screw fixation not only achieves comparable pain reduction but also delivers the added advantage of structural stabilization. This dual benefit positions the technique as a superior alternative in certain clinical scenarios.

Balling et al. previously reported a reduction in the mean (SD) VAS scores from 8.9 (1.1) preoperatively to 3.6 (1.7) at discharge, with further improvement to 1.8 (1.9) at follow-up [2]. These results are consistent with the pain relief observed in the present cohort, despite demographic variations between study populations. The current study further expands on these findings by emphasizing the marked reduction in opioid use, a crucial metric in the context of pain management [27].

From a surgical perspective, the mean operative time of 55 min in this study is shorter than that reported for most navigated techniques, which often exceed 60–90 min [16,28]. Additionally, the observed screw loosening rate of 3.8% was notably lower than previously reported rates, potentially attributable to the frequent use of three screws for enhanced stability.

Our approach differed from a study conducted at the Department of Neurosurgery in Mainz [16], where two transsacral screws were the norm. We frequently utilized three screws and occasionally employed bilateral screw placement for efficiency. Our operation times were comparable, suggesting a similar level of efficacy. The range of hospital stay (3 to 25 days) reflected the heterogeneity of patient recovery: a few patients were able to be transferred to rehabilitation or discharged home very early (within 3–4 days) once pain was sufficiently managed, whereas others required prolonged inpatient care up to 3–4 weeks due to complications or slower mobilization progress. This variability is inherent in an elderly, comorbid population and underscores why we analyzed predictors of prolonged stay.

While several studies [16,21,22,28] have focused on the precision of screw placement using navigation [17,18,24,28], they did not delve into patient outcomes like pain reduction. Our study bridges this gap, suggesting that the precision afforded by navigation translates into tangible clinical benefits, including less tissue trauma, reduced blood loss, and quicker recovery.

Preliminary financial analyses indicate a positive financial balance with reduced surgery times [29]. However, the suitability of minimally invasive spine surgery varies based on patient condition, health status, surgical expertise, and cost of operating room time and personnel. The advantages of 3D navigation over traditional techniques can be inferred from broader trends in minimally invasive surgery. Additionally, the amount of radiation exposure for the operation team is reduced.

### 4.4. Limitations and Future Directions

While the results are compelling, certain limitations must be acknowledged. Most patients were in their seventh to ninth decades, but no fixed age threshold was required for inclusion; eligibility was determined by clinical and imaging criteria for sacral insufficiency fracture. Sacral insufficiency fractures often occur in osteoporotic bone; however, only 52.8% of our cohort had a formal osteoporosis diagnosis, so we focused on imaging-confirmed SIF rather than requiring documented osteoporosis in every case. Moreover, our retrospective design lacks a control group (such as a cohort managed conservatively or treated with sacroplasty), which limits the strength of any comparative conclusions. We cannot definitively say that navigated screw fixation is superior to other treatments based on this study alone. Future research should include prospective or randomized controlled trials comparing these interventions to validate our findings and to establish evidence-based guidelines.

The follow-up duration of three months provides valuable insights into early postoperative recovery but falls short of evaluating long-term outcomes such as screw integrity, delayed complications, and fracture nonunion rates. Extended follow-up periods exceeding 12 months are needed to address these gaps and ascertain the durability of the technique. Additionally, we recognize that standardized patient-reported outcome measures (e.g., EQ-5D and SF-36) were not included in this retrospective series, as they were not part of routine clinical protocols during the study period. This omission limits our ability to evaluate health-related quality of life and patient satisfaction following navigated transsacral fixation. We therefore recommend that future prospective investigations incorporate validated patient-reported instruments to capture these important dimensions.

Selection bias is another limitation, as the study cohort consisted solely of patients who failed conservative management. A broader, multi-center study encompassing a wider spectrum of patient demographics and clinical presentations would enhance the generalizability of the results.

Finally, the 3D navigation equipment and training required for this technique present significant upfront costs. Not all centers, especially smaller or resource-limited hospitals, may have access to such technology. We acknowledge that the cost–benefit balance of navigated fixation needs to be addressed in future studies. Investigations into cost-effectiveness should accompany clinical trials to determine if the improved outcomes (e.g., reduced length of stay or complications) justify the additional resource utilization.

## 5. Conclusions

The 3D-navigated transsacral screw fixation is a promising, minimally invasive technique for the treatment of sacral insufficiency fractures. It provides rapid and significant pain relief, enables early mobilization in most patients, and can reduce reliance on opioid analgesics. The low incidence of complications and reoperations further underscores its safety and effectiveness. By overcoming the limited stability of conservative management and the risks of non-navigated fixation, this approach offers a targeted solution for patients who fail nonoperative care. The observed improvements in pain management, functional recovery, and resource efficiency underscore the potential of this technique. However, these findings should be validated in prospective studies before broadly adopting the technique. Further research should also explore long-term outcomes and refine patient selection criteria to optimize its impact on patient care.

## Figures and Tables

**Table 1:** Demographic, clinical, and fracture characteristics of the study population.

Variable		n = 53
Female ^1^		43 (81.1)
Age (years) ^2^		78.7 (10.8) 43.7–92.4
BMI (kg/m^2^) ^2^ n = 52		24.9 (4.2) 16.4–35.3
Comorbidities ^1^	Osteoporosis	28 (52.8)
	DMT2	5 (9.4)
	HTA	28 (52.8)
	CHF	2 (3.8)
	CHD	3 (5.7)
	COPD	4 (7.5)
	CKD	1 (1.9)
N° of comorbidities ^3^		5 (5) 1–16
Minor trauma ^1^ n = 50		23 (46)
Symptom duration (days) ^3^ n = 52		16 (34) 1–274
FFP classification ^1^ n = 51	IIa	31 (60.8)
	IIc	6 (11.8)
	IIIb	1 (2.0)
	IIIc	2 (3.9)
	IVb	11 (21.6)
OF-Pelvis classification ^1^ n = 52	OF1	24 (46.2)
	OF2	1 (1.9)
	OF3	6 (11.5)
	OF4	21 (40.4)

^1^ Presented as frequency (percentage). ^2^ Presented as mean (standard deviation) and range (min–max). ^3^ Presented as median (interquartile range) and range (min–max). Abbreviations: BMI: body mass index; DMT2: diabetes mellitus type 2; HTA: hypertension; CHF: congestive heart failure; CHD: coronary heart disease; COPD: chronic obstructive pulmonary disease; CKD: chronic kidney disease; FFP: Fragility Fracture of the Pelvis; OF-Pelvis: Osteoporotic Fracture of the Pelvis.

**Table 2:** Surgical procedure characteristics.

Variables		n = 53
Surgery levels ^1^	S1	1 (1.9)
	S1 + S2	52 (98.1)
Total transsacral screws ^1^	None	3 (5.7)
	One screw	13 (24.5)
	Two screws	17 (32.1)
	Three screws	20 (37.7)
Total unilateral screws ^1^	None	29 (54.7)
	One screw	3 (5.7)
	Two screws	12 (22.6)
	Three screws	5 (9.4)
	Four screws	4 (7.5)
Screw correction ^1^		1 (1.9)
Surgery duration (minutes) ^2^		55 (24) 31–145

^1^ Presented as frequency (percentage). ^2^ Presented as median (interquartile range) and range (min–max).

**Table 3:** Assessment of pain outcomes and presence of paresis.

Variables	Pre-Surgery n = 53	Post-Surgery n = 53	1 Month n = 53	3 Months n = 53	*p*-Value	Effect Size
Opioid requirement ^1^	47 (90.4) n = 52	49 (92.5) n = 52	19 (51.4) n = 37	9 (45.0) n = 37	0.003	0.21
Paresis ^1^	4 (7.5)	3 (5.7)	0 (0.0) n = 46	2 (3.8)	NS	0.01
Cumulated Ambulation Score ^2^	2 (1) 0–3	2 (0) 0–3 n = 53	2 (1) 0–3 n = 32	2 (0) 0–3 n = 33	NS	0.01

^1^ Reported as frequency (percentage). *p*-value determined through Cochran’s Q test and Kendall’s W was calculated to quantify effect size. ^2^ Reported as median (interquartile range) and range (min–max). *p*-value was determined through Friedman test for related samples for two-factor analysis of variance by ranks; partial eta squared was calculated to determine the effect size. Abbreviations: VAS: Visual Analog Scale (0–10 pain scale); NS: not significant (*p* > 0.05).

**Table 4:** Hospital stay and complication characteristics.

Variables		n = 53
Surgery complications ^1^	Bleeding	1 (1.9)
	Wound infection	3 (5.7)
	CSF leak	0 (0.0)
	New neurologic deficit	0 (0.0)
Start of mobilization ^1^	Day 1	51 (96.2)
	Day 2	1 (1.9)
	After day 2	1 (1.9)
Hospital stay (days) ^2^		11.7 (5.1) 3–25
Screw alterations (at 3 months) ^1^ (n = 52)	None	45 (86.5)
	Loosening	2 (3.8)
	Backed-out	5 (9.4)
Reoperations (at 3 months) ^1^ (n = 52)		4 (7.7)

^1^ Presented as frequency (percentage). ^2^ Presented as mean (standard deviation) and range (min–max). Abbreviations: CSF: cerebrospinal fluid.

## Data Availability

The data supporting the findings of this study are available from the corresponding author upon reasonable request. Due to ethical and legal considerations, the data are not publicly accessible.

## Supplementary Materials

The following supporting information can be downloaded at: https://www.mdpi.com/article/10.3390/jcm14113749/s1, Video S1: Procedural Steps.
